# Using a Concept Map as a Tool for Strategic Planning: *The Healthy Brain Initiative*

**Published:** 2011-08-15

**Authors:** Lynda A. Anderson, Kristine L. Day, Anna E. Vandenberg

**Affiliations:** Healthy Aging Program, Division of Adult and Community Health, Centers for Disease Control and Prevention. Dr Anderson is also affiliated with the Department of Behavioral Sciences and Health Education, Rollins School of Public Health, Emory University, Atlanta, Georgia.; Healthy Aging Program, Division of Adult and Community Health, Centers for Disease Control and Prevention, Atlanta, Georgia; Healthy Aging Program, Division of Adult and Community Health, Centers for Disease Control and Prevention, Atlanta, Georgia

## Abstract

Concept mapping is a tool to assist in strategic planning that allows planners to work through a sequence of phases to produce a conceptual framework. Although several studies describe how concept mapping is applied to various public health problems, the flexibility of the methods used in each phase of the process is often overlooked. If practitioners were more aware of the flexibility, more public health endeavors could benefit from using concept mapping as a tool for strategic planning.

The objective of this article is to describe how the 6 concept-mapping phases originally outlined by William Trochim guided our strategic planning process and how we adjusted the specific methods in the first 2 phases to meet the specialized needs and requirements to create *The Healthy Brain Initiative: A National Public Health Road Map to Maintaining Cognitive Health*. In the first stage (phases 1 and 2 of concept mapping), we formed a steering committee, convened 4 work groups over a period of 3 months, and generated an initial set of 42 action items grounded in science. In the second stage (phases 3 and 4), we engaged stakeholders in sorting and rating the action items and constructed a series of concept maps. In the third and final stage (phases 5 and 6), we examined and refined the action items and generated a final concept map consisting of 44 action items. We then selected the top 10 action items, and in 2007, we published *The Healthy Brain Initiative: A National Public Health Road Map to Maintaining Cognitive Health, *which represents the strategic plan for The Healthy Brain Initiative.

## Introduction

Strategic planning is recognized as an essential component of public health endeavors (1). Strategic planning can assist in assessing what has been done and what can and should be done. In 2005, Congress appropriated funds to the Centers for Disease Control and Prevention (CDC) to address cognitive health (2). That same year, the Centers for Disease Control and Prevention and the Alzheimer's Association formed a new partnership, The Healthy Brain Initiative, to examine how best to bring a public health perspective to the promotion of cognitive health. The partnership recognized a need to develop a strategic plan to identify public health priorities, create a unified vision among stakeholders, and guide activities over a 3- to 5-year period.

Concept mapping is a tool that helps with strategic planning. It consists of a sequence of phases that result in a conceptual framework (3). A concept map provides a visual picture of strategic planning ideas; the ideas are clustered in groups so that a complex set of ideas can be more readily understood. Concept mapping has taken various forms, such as "idea mapping" or "mind mapping," to enhance creative thinking or improve the organization of ideas (4). Concept mapping for public health, a process introduced by William Trochim (3), involves a group of stakeholders who have an interest in a given area (eg, researchers, practitioners) or may be affected by the outcomes (eg, community members, program participants). Concept mapping has been applied to create logic models for a national program (5), develop various state plans (6,7), and design chronic disease competencies (8). Articles describing these concept maps focus on the outcome of the mapping process and often, as a result, overlook the flexibility of the methods at each phase (9). More public health endeavors could benefit from concept mapping as a strategic planning tool if practitioners were more aware of the flexibility of the methods at each phase of the process.

The objective of this article is to present an overview of concept mapping to public health practitioners and to show how phases of the concept-mapping process can be altered or tailored to meet special requirements. To illustrate concept mapping in concrete terms, we show how the tool was applied to create a conceptual framework for *The Healthy Brain Initiative: A National Public Health Road Map to Maintaining Cognitive Health* (10), hereafter referred to as the Road Map. In creating the Road Map, we altered the first 2 phases of Trochim's original phases to meet our own requirements. One requirement was to align action items in the Road Map with current science. Another was to incorporate input from a broad group of stakeholders, including content experts, practitioners, and policy makers. A participatory process was important because a broad group of stakeholders enhances the perceived validity of a framework (11). Another modification was the use of multiple modes of communication over several months instead of a single brainstorming session.

## Development of the Concept Map

The state of the science of cognitive health (12) and growing concerns about cognitive impairment shaped the strategic planning process for bringing a public health perspective to the promotion of cognitive health (13). In May 2006, The Healthy Brain Initiative hosted a meeting of national experts to review research and discuss recommendations for promoting cognitive health; participants focused on vascular risk factors and physical activity (14). Findings from this meeting provided a foundation for The Healthy Brain Initiative's next step: developing a strategic plan.

**Box.** Phases of Concept Mapping as Originally Conceptualized by Trochim (3)

**Phase 1: Preparation**
Planning group identifies a single focal question or prompt that will best serve the goal of the project.
Planning group identifies participants to generate ideas through brainstorming.
**Phase 2: Generation of Ideas**
Participants brainstorm ideas in a single in-person session or online.
Planning group selects core group of ideas from ideas generated by participants (no more than 100).
**Phase 3: Structuring of Statements**
Planning group identifies and invites external participants to sort and rate the core group of items.
Participants sort and rate the core group of items.
**Phase 4: Representation of Statements**
Consultants or staff compute a series of maps using concept-mapping software (multidimensional scaling and cluster analysis).
**Phase 5: Interpretation of Maps**
Planning group examines the point and cluster maps, generates a final cluster map, and agrees on a descriptive phrase or word that captures the meaning or essence of each cluster.
**Phase 6: Use of Maps**
Planning group relates maps and associated materials to the original goal of the project and produces plan for further action.

We organized the strategic planning process into 3 overarching stages using Trochim's 6 concept-mapping phases (Box) as a guide. The first stage (including Trochim's phases 1 and 2), project planning, consisted of the formation of a steering committee and work groups and preparation and generation of statements, or action items. The second stage (including Trochim's phases 3 and 4), generating concept maps, consisted of the structuring and representation of action items, including sorting and rating action items and constructing a series of concept maps. The third phase (including Trochim's phases 5 and 6), finalizing the framework, consisted of the interpretation and use of maps. In this final phase, the steering committee examined and refined the concept maps, labeled the clusters, created 2 new items, and selected the top 10 action items for the Road Map.

### Stage 1: Project planning

**Phase 1: Preparation**. We formed a 12-member steering committee and established 4 work groups. Steering committee members represented varied disciplines and sectors in public health and cognitive health. The steering committee guided the overall concept-mapping process, assisted with identifying and recruiting members of work groups, helped define the charge to the work groups, participated in generating the concept maps, helped to interpret and finalize the concept map, and determined the final set of 10 priority action items. The CDC co-leader of the steering committee (L.A.A.) took primary responsibility for formulating the planning process, and CDC hired a project manager (K.L.D.) who coordinated logistics for the work groups and communication between the work groups and steering committee.

Consistent with a typical concept-mapping process, the steering committee developed a charge, similar to a "focus prompt,"' to articulate the goal of the concept-mapping process. The charge was to "develop a set of recommended actions for moving the nation forward over the next 3 to 5 years toward the long-term goals of maintaining and improving the cognitive function of adults."

Also consistent with a typical concept-mapping process, the steering committee identified and recruited people to work groups, which served as vehicles for generating ideas. Work groups were established for 4 content areas: prevention research, surveillance, communication, and policy. The Healthy Brain Initiative required that ideas and action items be grounded in science, so the steering committee developed criteria to identify eligible participants. For example, the criteria for the prevention research work group included people with experience in phase 2 translational research (from clinical studies to community-level interventions), translational research from other successful areas (eg, diabetes, physical activity, cardiovascular health), community-based interventions, research measurement, study design, and community-based participatory research. Work group members represented varied sectors (eg, nonprofit, government, academia), disciplines (eg, epidemiology, gerontology, social work), and perspectives (eg, aging, public health, policy). Each work group consisted of no more than 20 participants, and each group was asked to develop an initial set of action items.

**Phase 2: Generation of statements, or ideas.** Using an iterative process, the 4 work groups worked independently over 3 months to generate a set of action items. Each work group selected 2 members to facilitate discussions and draft the rationale statements that accompanied each action item. In addition, each work group developed a definition for its content area (eg, prevention research) and identified key principles and audiences. In a departure from the typical concept-mapping process, which relies on a single online or in-person brainstorming session, each group participated in multiple conference calls and corresponded through e-mail between calls.

Before finalizing its list of action items, each work group sent its list to a group of external reviewers to determine whether items were understandable and to identify any missing items. Finalized lists were submitted to the steering committee. The prevention research work group generated 18 action items (eg, "Conduct controlled clinical trials to determine the effect of physical activity on reducing the risk of cognitive decline and improving cognitive functions"). The policy work group generated 9 action items (eg, "Include cognitive health in *Healthy People 2020*, a set of health objectives for the nation that will serve as the foundation for state and community health plans"). The communication work group generated 8 action items (eg, "Determine how diverse audiences think about cognitive health and its associated risk factors"). The surveillance work group generated 7 action items (eg, "Determine a population-based surveillance system with longitudinal follow-up that is dedicated to measuring the public health burden of cognitive impairment in the United States").

### Stage 2: Generating concept maps

Throughout the first stage, the work groups worked independently of one another. This second stage of the process allowed a larger group of participants to work collectively to unite the varied action items into a cohesive framework or common vision. It also allowed the steering committee to understand how the entire group of stakeholders collectively rated the importance and action potential of the items.

**Phase 3: Structuring of statements**. The first step in Phase 3 was to recruit a larger group of people to sort and rate all the action items identified by the work groups. The steering committee enlisted a contractor, Concept Systems Incorporated, which restructured the 42 items for their proprietary software tool. The tool allowed participants independently and anonymously to sort and rate the items on the project website. We invited 31 people, including steering committee members and 19 additional people representing the fields of cognition, aging, and public health, to participate in sorting. Using the software online, participants were asked to create their own categories. They were instructed to place each statement into only 1 category. The instructions also stated that the sorting process should result in more than 1 category but fewer categories than the total number of statements.

The steering committee asked a second larger group of 141 people, including 21 from the sorting task, to rate the items. Participants included members of the steering committee and work groups and external reviewers. Participants rated each item on 2 dimensions: importance ("How important the item was to a cognitive public health agenda") and action potential ("How feasible the implementation of the idea would be"). The items appeared in random order on the project website. Because participation was anonymous, we could not calculate exact response rates. However, on the basis of unique identifiers, we estimated that 83% of the 31 stakeholders participated in sorting, and about 49% of the 141 rated the items. These rates are comparable to other concept-mapping projects (5).

**Phase 4: Representation of statements**. This phase involves the computation of a series of concept maps. We generated the concept maps using Concept Systems software 4.0 (Concept Systems Incorporated, Ithaca, New York) using methods developed by Trochim (3). First, the software assigns a unique number to each action item, assesses the number of sorting participants who categorized action items similarly, and then generates an aggregate similarity matrix. Second, the software analyzes the aggregate similarity matrix by using multidimensional scaling analysis and for each action item, generates *x* and *y* coordinates in 2-dimensional space (15). Third, the software combines action items into clusters using hierarchical cluster analysis (16). Next, the software superimposes the results of the hierarchical cluster analysis on the multidimensional scaling analysis, creating a point map. Finally, the software creates an initial cluster map by placing boundaries around the items that make up a cluster. Clusters are initially made up of about 5 items, but the software allows for fewer or greater numbers of items in each cluster. This concept map should be considered the initial solution, however, because it is the starting point for reviewing the findings and determining the final cluster map (4).

Action items depicted together in cluster are more similar to one another than they are to items in other clusters. Items that appear closer together in a cluster are more similar to one another than they are to items farther apart. Likewise, clusters that are closer together on the map contain items that are more similar to the items in near clusters than they are to clusters farther apart. The overall size of a cluster reflects how similar or correlated the items are to each other as well as the number of items in a cluster. Concept maps have no top or bottom. In other words, the orientation of the clusters relative to the top or bottom of the map has no particular meaning.

### Stage 3: Finalizing the framework

**Figure 1:**
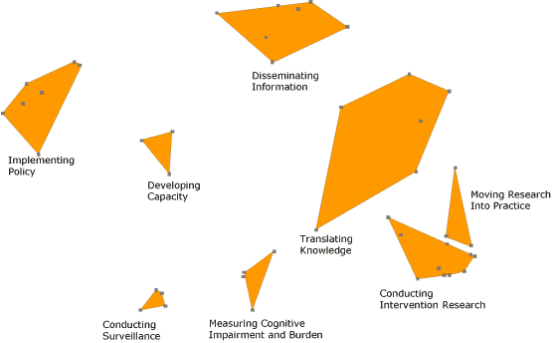
Final concept map that served as the framework for *The Healthy Brain Initiative: A National Public Health Road Map to Maintaining Cognitive Health *(10). Points on the map represent the items as entered in the concept-mapping software. Items within a cluster are more similar to one another than they are to items in other clusters. The number of action items in a cluster and their similarity determine the shape, boundaries, and size of each cluster.

**Phase 5: Interpretation of maps**. As in a typical concept-mapping process, the steering committee examined the maps and made several changes. The committee created 2 new action items by reconstructing existing items, and it moved several action items from 1 cluster to another. The software consultants subsequently reran the analyses, moved additional items, and produced a final concept map with 8 clusters and 44 action items (Figure 1). The steering committee agreed upon cluster names. The cluster "Developing Capacity" originated exclusively from action items from the prevention research work group. "Implementing Policy" items originated exclusively from the policy work group, "Conducting Surveillance" items originated exclusively from the surveillance work group, and "Intervention Research" items originated exclusively from the prevention research work group. All other clusters were formed from various action items derived from several work groups. This final concept map served as the organizational framework for the Road Map.

**Table:** 

Figure 1 shows the final cluster map for the The Healthy Brain Initiative (10). The figure shows 8 clusters, which vary in size and shape. Each cluster is made up of a series of points that represent the individual action items generated by the work groups and reformatted for the concept-mapping software. Items in a cluster are more similar to one another than they are to items in other clusters. The size of a cluster reflects the similarity of the items and the number of items in the cluster. How close together the clusters appear on the map reflects how similar the items are in those clusters. Seven of the clusters are located on the periphery of the graphic, forming an elliptical circle. Only 1 cluster, “Developing Capacity,” is surrounded completely by others.
Starting with the cluster labeled “Disseminating Information” (placed at the top) and moving clockwise, the next cluster is “Translating Knowledge.” The next set of 2 clusters, appearing closely together at the right, is “Moving Research into Practice” and “Conducting Intervention Research.” At the bottom, directly opposite the top cluster, is “Measuring Cognitive Impairment and Burden.” To the left is “Conducting Surveillance.” Moving directly up is the most central of the clusters, “Developing Capacity.” The eighth cluster, to the far left, is “Implementing Policy.”

**Figure 2:**
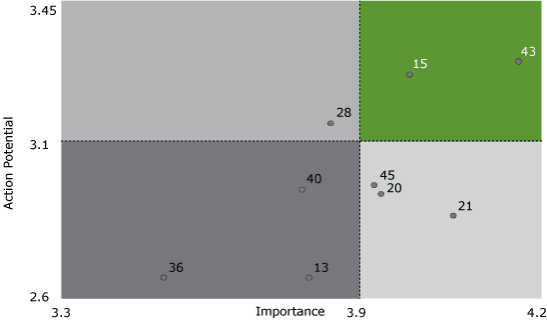
A sample go-zone analysis for 1 of the clusters, "Implementing Policy," in the final concept map for *The Healthy Brain Initiative: A National Public Health Road Map to Maintaining Cognitive Health* (10). Each dot represents an action item and is identified by a unique number. Items were scored for importance (from 1, relatively unimportant, to 5, extremely important) and action potential (from 1, no action potential, to 4, high action potential) during a rating process. The upper right quadrant, or go-zone, highlighted in green, displays items rated as most actionable and important.

**Phase 6: Use of maps**. In this phase, as in a typical concept-mapping process, we accomplished the original goal of the project, which was to create a strategic plan, a set of recommended actions for the next 3 to 5 years. We identified this set of actions by using data from the rating process to construct *go-zones*, a visual display of action items rated as most actionable and important. The go-zone for the cluster "Implementing Policy" includes 2 priority items (Figure 2).

**Table:** 

Item Number	Items Included in the Rating Process From the Cluster "Implementing Policy"
13	Develop creative and replicable means for raising public awareness about and engaging the public in promoting the importance of cognitive health through policy.
15	Develop and implement a strategy to have cognitive health included in *Healthy People 2020*.
20	Identify and promote appropriate strategic partnerships among associations, government agencies, insurers and payers, private industry, public organizations, elected officials to support and advance policy related to cognitive health.
21	Educate federal, state, and local officials responsible for addressing issues concerning the older population, lifestyle factors, or diseases/conditions related to cognitive health to initiate and support policy changes to promote cognitive health.
28	Engage national organizations/agencies that focus on the older populations, and educate these agencies about cognitive health and its connection to the mission of their organization.
36	Develop and implement a strategy to include subjects related to cognitive health in curricula for continuing professional education of health and human services professionals.
40	Convene policy experts to identify and examine current policies (eg, national policy, state policy, private sector policy) that could be modified, modernized, or broadened to include cognitive health.
43	Include cognitive decline in the *State of Aging and Health in America* report when population-level data are available.
45	Promote the modification of existing national and state public health plans that address key health issues related to cognitive health to include cognitive health in their strategies or recommendations where appropriate.

The steering committee reviewed the go-zone analysis for each of the 8 clusters, identified potential priority items, revised some wording, and selected a final set of 10 priority action items from 6 clusters:

- Disseminate the latest science to increase public understanding of cognitive health and to dispel common misconceptions (from the cluster "Disseminating Information").
- Determine how diverse audiences think about cognitive health and its associations with lifestyle factors ("Translating Knowledge").
- Help people understand the connection between risk and protective factors and cognitive health ("Translating Knowledge").
- Conduct systematic literature reviews on proposed risk factors (vascular risk and physical activity) and related interventions for relationships with cognitive health, harms, gaps and effectiveness ("Moving Research Into Practice").
- Conduct controlled clinical trials to determine the effect of reducing vascular risk factors on lowering the risk of cognitive decline and improving cognitive function ("Conducting Intervention Research").
- Conduct controlled clinical trials to determine the effect of physical activity on reducing the risk of cognitive decline and improving cognitive function ("Conducting Intervention Research").
- Conduct research on other areas potentially affecting cognitive health such as nutrition, mental activity, and social engagement ("Conducting Intervention Research").
- Develop a population-based surveillance system with longitudinal follow-up that is dedicated to measuring the public health burden of cognitive impairment in the United States (from the cluster "Conducting Surveillance").
- Initiate policy changes at the federal, state, and local levels to promote cognitive health by engaging public officials ("Implementing Policy").
- Include cognitive health in *Healthy People 2020*, a set of health objectives for the nation ("Implementing Policy").

On the basis of results of the concept-mapping process, we designed the Road Map (10) and disseminated it to more than 1,000 dementia experts at the 2007 Alzheimer's Association International Conference on Prevention of Dementia in Washington, DC. The Road Map appears on CDC's Healthy Aging website (http://www.cdc.gov/aging/healthybrain/index.htm), on many partner websites, and it has been cited in numerous publications and grants. The Healthy Brain Initiative relies on the Road Map to identify what actions to pursue and how to best collaborate with other partners that share an interest in those actions (17). CDC uses the 10 priority actions as a means to communicate and support activities (18).

## A Flexible Process

A chief advantage of concept mapping is its flexibility, which allows users to refine ideas and the process itself. This flexibility allowed us to tailor the process to combine 2 separate but equally important approaches. One approach was to elicit action items from content experts independently. Independent submission of ideas across the areas of research, surveillance, policy, and communication strengthened the validity of the action items. The second approach was participatory: a diverse group of stakeholders collectively rated and sorted all action items to develop a cohesive framework. In an additional modification, we generated initial ideas through 4 work groups that communicated by e-mail and conference call over 3 months instead of relying on a single brainstorming session. The entire process — from the formation of the steering committee to the publishing of the Road Map — took approximately 18 months.

We encountered 2 major challenges in developing the Road Map. First, it took more work than expected to structure the action items submitted by the work groups into a form that was acceptable for the concept-mapping software. Second, steering committee members identified and refined several action items that could not be included in the final map because the items were not included in the sorting and rating process. Despite these challenges, concept mapping provided a structured process that allowed for flexibility in a way that best suited our needs. We were able to engage a diverse group of stakeholders, manage a large amount of information, and frame a complex set of interrelated ideas.

The process allowed for different types of participants, numbers, and types of focus questions, ways of sorting and rating, and interpretations and uses. As Trochim indicated, "The uses of the map are limited only by the creativity and motivation of the group" (3). Future research on concept maps could help to articulate the complete range of options for methods, measures, and analyses.
